# Genotypic and Phenotypic Characterization of Antimicrobial-Resistant *Escherichia coli* from Farm-Raised Diarrheic Sika Deer in Northeastern China

**DOI:** 10.1371/journal.pone.0073342

**Published:** 2013-09-09

**Authors:** Rui Li, Liang He, Lili Hao, Qi Wang, Yu Zhou, Hongchen Jiang

**Affiliations:** 1 State Key Laboratory Breeding Base for Zhejiang Sustainable Plant Pest Control, Agricultural Ministry Key Laboratory for Pesticide Residue Detection, Institute of Quality and Standard for Agro-products, Zhejiang Academy of Agricultural Sciences, Hangzhou, Zhejiang, China; 2 Shanghai Institute of Pharmaceutical Industry, National (Shanghai) Center for New Drug Safety Evaluation and Research, Shanghai, China; 3 College of Life Science and Technology, Southwest University for Nationalities, Chengdu Sichuan, China; 4 Animal Nutrition and poultry Science, Guelph University, Guelph, Ontario, Canada; 5 State Key Laboratory of Geobiology and Environmental Geology, China University of Geosciences, Wuhan, Hubei, China; University of Birmingham, United Kingdom

## Abstract

In China, overuse and/or abuse of antimicrobials are common in stockbreeding, which possess high risks of antimicrobial-resistant contaminations. The serogroups, major virulence genes, and antimicrobial resistant patterns of the antimicrobial-resistant *Escherichia coli* (*E. coli*) were investigated in the feces of diarrheic farm-raised sika deer from 50 farms in three Northeastern provinces of China. A total of 220 *E. coli* isolates were obtained and characterized. Twenty-eight O serogroups were identified from the obtained *E. coli* isolates with O2, O26, O128, O142 and O154 being dominant. Nearly all the isolates were resistant to at least four of the tested antimicrobials. More than 90% of the *E. coli* isolates carried at least one of the tested virulence genes. About 85% of the *E. coli* isolates carried one or more antimicrobial-resistant genes responsible for resistant phenotypes of sulfonamides, streptomycin/spectionomycin or tetracycline. The antimicrobial resistant level and pathogenic group occurrences of the obtained *E. coli* isolates were higher than that of livestock and wild animals reported in some developed countries. Thus, the fecal-carrying antimicrobial-resistant *E. coli* from the farm-raised sika deer is potentially a significant contamination source for freshwater systems and food chain, and may pose great health risks for human and animals in Northeastern China.

## Introduction

Antimicrobial resistance (AMR) in enteropathogens has become a major public health problem due to its potential infections on human and animals [1]. The domestic animals are usually considered as major reservoirs for antimicrobial-resistant bacteria. Recently, increasing interest has been given to antimicrobial-resistant pathogenic bacteria from various domestic animals and their habitats. Antimicrobial-resistant bacteria have been detected in a variety of domestic animals and the environments that are affected by stockbreeding [2], [3]. *E. coli*, a type of bacteria common in the intestine of warm-blooded animals, was widely used as an indicator of fecal contamination in drinking water system assessment and food safety evaluation [4]. Pathogenic *E. coli* is an important pathogen that can infect humans and animals. Various pathotypes of *E. coli* can be distinguished by the virulence genes [5]. Infection by pathogenic *E. coli* mainly cause diarrhea in domestic livestock, especially in young animals with clinical syndromes including acute severe watery diarrhea, haemorrhage, and sudden death [6]. Among the identified pathotypes, EPEC (enteropathogenic *E. coli*), ETEC (enterotoxigenic *E. coli*), and STEC (Shiga-like toxin-producing *E. coli*) strains represented three major classes of enteric pathogens leading to diarrhoea in humans and animals [7]. Previous studies showed that feces from wild deer could contaminate surface water that may be used as drinking water for humans and/or domestic animals [8]. Thus, the pathogenic *E. coli* strains from domesticated wild animals (e.g., domesticated wild deer) can also be transmitted to humans [9], [10].

As domesticated wild animals, China has a large population of farm-raised sika deer (*Cervus nippon*), mainly distributed in three Northeastern provinces (Liaoning, Jilin and Heilongjiang) with about 80% sika deer production in China [11]. Although the farming pattern of farm-raised sika deer is similar to the domestic ruminants (e.g., cattle, sheep); there are still some uniqueness in its farming production. For example, the mainly purpose of farm-raised sika deer in China is to obtain pilose autler rather than for meat production, and the feeding cycle of farm-raised sika deer is usually longer than two years. Therefore, antimicrobials are not added (or only a small amount of antimicrobials are added) into daily feed, but they are used heavily when disease outbreaks. In comparison, the mainly purpose of domestic ruminants in China are meat production with a feeding cycle of less than one year; excessive antimicrobials are applied in the daily feed for improving growth performance. Compare to wild animals (including wild deer), the diarrheic farm-raised deer accepting heavy amounts of antimicrobials for disease treatment may be a larger reservoir of potential AMR pathogens. However, to our knowledge, few studies have been focused on AMR pathogens of farm-raised diarrheic sika deer and their potential risk to public health. The objective of this study is to characterize the diversity of serological types, the distribution of antimicrobial-resistant patterns and virulence genes of AMR *E. coli* in the feces from farm-raised diarrheic sika deer in Northeastern China.

## Results and Discussion

### Serogroup differences between farm-raised sika deer and other wild animals/domestic ruminants

**Figure 1:**
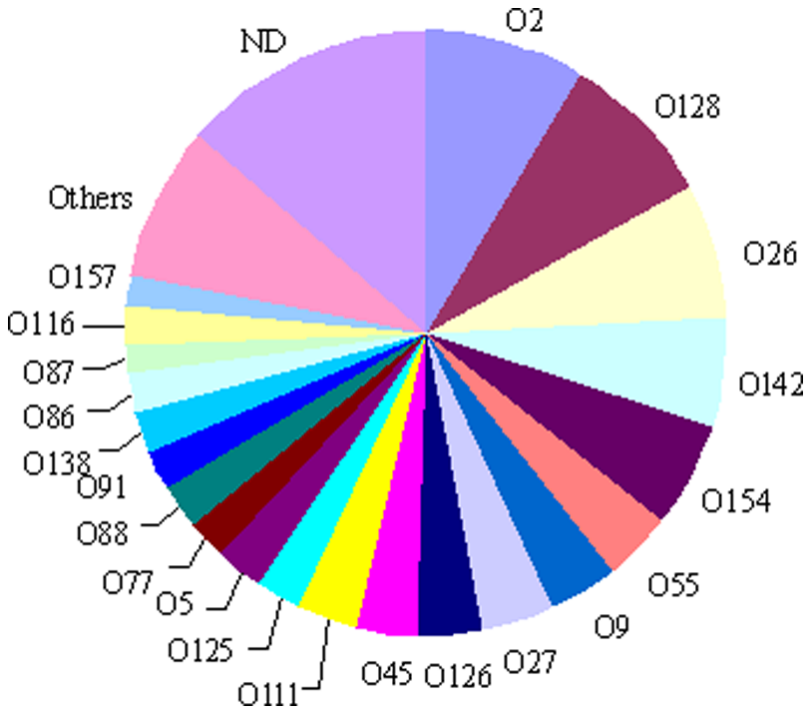
O serogroups distribution among 220 *E. coli* isolates from farm-raised sika deer sourced from three Northeastern provinces of China. Note: Others refer to O20, O25, O32, O44 (three isolates of each), and O6, O8, O103 (two isolates of each); ND refers to unknown O serogroups.

In this study, 10 isolates with morphology of *E. coli* were randomly picked up from each deer farm (also from each fecal sample) and subjected to biochemical identification. Among a total of 500 suspect bacterial isolates, 220 of them were identified as typical *E. coli* strains. One hundred ninety of the obtained *E. coli* isolates were classified into 28 different types of O serogroups, and the remaining did not belong to any known serogroups (untypable or O-rough) (Figure 1). Around 60% of the identified O serogroups belonged to twelve major groups: O2, O128, O26, O142, O154, O55, O9, O27, O126, O45, O111, and O125, with the former five O serogroups being dominant. Four *E. coli* isolates were affiliated with serogroup O157, which is known to be associated with life threatening diseases [10].

More than a half of the identified O serogroups in this study have never been reported from wild deer to date, and a few peculiar O serogroups identified here (e.g., O9, O26, O138 and O157) might be associated with human and livestock infections [5], [12], [13]. Prevalence rate of *E. coli* O157 from diarrheic farm-raised sika deer was 1.8%, which was significantly higher than 0.3% and 0.25% of wild animals (including wild deer) in Louisiana and Nebraska USA, respectively [14], [15]. This indicated that farm-raised sika deer has become natural reservoir for pathogenic *E. coli* O157 in Northeastern China. The dominant serogroups of the *E. coli* isolates in this study were markedly different from those of diarrheic sheep flocks (O5, O6, O76, O87, O91, O123, O128, O146, O166 and O176) and cattle calves (O56, O78, O8 and O164) [3], [16]. The observed difference in serogroups among farm-raised sika deer, livestock and wild animals might be ascribed to their different feeding managements, habitats and antimicrobials ingests. The underlying reasons await further investigation.

### Occurrence of virulence genes and pathogenic *E. coli* groups in the farm-raised sika deer

The majority of the *E. coli* isolates in this study carried at least one of the twelve tested virulence genes, some of which occurred in high frequency (Table 1). A total of 74 (33.6%) isolates carried only one virulence gene, whereas 128 (58.2%) isolates carried more than one investigated virulence genes. The genes of *astA*, *eaeA*, *stx2*, *fasA*, *aidA-1*, *elt*, *estB*, *faeG*, and *stx1* were present in 134 (60.9%), 43 (19.5%), 42 (19.1%), 40 (18.2%), 28 (12.7%), 28 (12.7%), 24 (10.9%), 18 (8.2%), and 18 (8.2%) of the *E. coli* isolates, respectively. The *estA* and *fedA* genes were present in less than 2.0% of the *E. coli* isolates, while the *fanC* gene was not detected. The pathogenic potential of *E. coli* can be inferred based on virulence genes [5]. A total of 163 (74.1%) isolates were shown to carry at least one of the seven types of virulence genes (*eaeA*, *faeG*, *fasA*, *fedA*, *elt*, *estA*/*estB* and *stx1*/*stx2*). There were 43 (19.5%), 9 (4.1%), and 33 (15.0%) isolates carrying virulence genes of *eaeA* (EPEC), *stx1* (STEC) and *stx2* (STEC), respectively; 9 (4.1%) isolates carried virulence genes of *stx1* and *stx2* (STEC). A total of 69 isolates (31.4%) carried at least one of the ETEC virulence genes (*faeG*, *fasA*, *fedA*, *elt*, and *estA*/*estB*). The frequency of the LT *(elt)* and ST (*estA*/*estB*) genes was 6.8% in the *E. coli* isolates. About 26.0% (18 out of 69) and 2.9% (2 out of 69) of the ETEC isolates carried genes for F4 (*faeG*) and F18 (*fedA*) colonization antigens, respectively, which were proved to be the most important fimbrial adhesins of ETEC causing livestock diarrhoea [12]. None of the obtained isolates carried *eaeA* and *stx* (enterohemorrhage *E. coli*, EHEC) virulence genes.

**Table 1:** Distribution of O serogroups, major virulence genes and pathotypes of *E. coli* isolates from farm-raised diarrheic sika deer.

O serogroups	*aidA-1*		*eaeA*	*faeG*	*fanC*	*fasA*	*fedA*	*astA*	*elt*	*estA*	*estB*	*stx1*		*stx2*	pathotype
O2 (19)	1	19		–	–	–	–	17	–	–	–	–	–		EPEC (19)^a^
O5 (5)	1	–		–	–	1	–	1	1	–	–	2	2		STEC (1)
O6 (2)	–			1	–	1	–	–	2	–	2	–	–		ETEC (2)
O8 (2)	–	–			–	1	–	2	1	–	1	–			ETEC (2)
O9 (8)	–	–		4	–	2	–	4	4	–	1	–	–		ETEC (8)
O20 (3)	–	–		–	–	–	1	–	–	1	3		–		ETEC (3)
O25 (3)	–	–		–	–	–	–	3	1	–	2	–	–		ETEC (3)
O26 (4)	4	2			–	–	–	10	–		–	–	–		EPEC (4)
O26 (1)	–			–	–	1	–	–	–		–	–	–		ETEC (1)
O26 (11)	–	–			–	–	–	–		–	–	5	6		STEC (11)
O27 (4)	–	–		2	–		–	3	3	1	3	–	–		ETEC (4)
O27 (3)	–	3			–	–	–			–	–	–	–		EPEC (3)
O27 (1)	–	–			–	–	–	1	–	–	–	–	–		NA (1)^b^
O32 (2)	–	–		–	–	2	–	–	–			–	–		ETEC (2)
O32 (1)	–	–		–	–	–	–	–		–	–	–	–		NA (1)
O44 (3)	–	–		–	–	–	–	3	–	–	–	–	–		NA (3)
O45 (7)	6	–		–	–		–	6	–	–	–	–	7		STEC (7)
O55 (7)	4	–		–	–	–	–	8	–	–	–	6	1		STEC (7)
O55 (1)	–	1		–	–	–	–	–	–	–	–	–	–		EPEC (1)
O55 (1)	–	–		–	–	1	–	–	–	–	–	–	–		ETEC (1)
O77 (5)	–	–			–	–	–	1	–	–	–	–	–		NA (5)
O86 (4)	–	3		–	–	–	–	3	–	–	–	–	–		EPEC (3)
O87 (1)	–	–		–	–	1	–	–	–	–	–	–	–		ETEC (1)
O87 (1)	–	1		–	–	–	–	–	–	–	–		–		EPEC (1)
O87 (2)	–	–		–	–	–	–	2	–	–	–	–	–		NA (2)
O88 (4)	–	–		2	–	1	–	2	4	–	3	–	–		ETEC(4)
O88 (1)	–	–		–		–	–	1	–	–	–	–	–		NA (1)
O91 (5)	2	–		–	–	–	–	2	–	–	–	–	5		STEC (5)
O103 (2)	1	–		–	–	–	–	1	–	–	–	–	2		STEC (2)
O111 (4)	–			–	–	–	–	4	–	–	–	–	4		STEC (4)
O111 (1)	1	1		–	–	–	–	–	–	–	–	–	–		EPEC (1)
O111 (1)	–	–		–	–	1	–	–	–	–	–		–		ETEC (1)
O111 (1)	–	–		–	–	–	–	1	–		–	–	–		NA (1)
O116 (1)	–	–		–	–	1	–	1	–	–	–	–	–		ETEC (1)
O116 (3)	–	–		–	–	–	–	3	–	–	–	–	–		NA (3)
O125 (3)	–	–		–	–	–	–	3	–	–	–	1	3		STEC (3)
O125 (2)	–	–		–	–	2	–	–	–	–	–	–	–		ETEC (2)
O125 (1)	1	–		–	–	–	–	1	–	–	–	–	–		EPEC (1)
O126 (8)	–	–		4	–	6	–	4	2	–	1	–	–		ETEC (8)
O128 (7)	1	–		–	–	–	–	7	–	–	–	–	7		STEC (7)
O128 (2)	–	2		–	–	–	–	–	–	–	–	–	–		EPEC (2)
O128 (8)	–	–		5	–	4	–	4	4	2	4	–	–		ETEC (8)
O128 (1)	–	–		–	–	–	–	1	–	–	–	–	–		NA (1)
O138 (2)	–	–			–	–	–	2	–	–	–	–	–		NA (2)
O138 (3)	–	–		–	–	–	–	–	–	–	–	–	–		NA (3)
O142 (8)	2	8		–	–	–	–	1	–	–	–	–	–		EPEC (8)
O142 (1)	–	–		–	–	1	–	–	–	–	–	–	–		ETEC (1)
O142 (4)	–	–		–	–	–	–	1	–	–	–	–	–		NA (4)
O154 (5)	–	–		–	–	5	–	2	1	–	–	–	–		ETEC (5)
O154 (7)	–	–		–	–	–	–	3	–	–	–	–	–		NA (7)
O157 (4)	2	–		–	–	–	–	3	–	–	–	4	–		STEC (4)
O?	1	–		–	–	1	1	6	–	–	1	–	5		STEC (7)
O?	–	–		–	–	8	–	10	5	–	3	–	–		ETEC (12)
O?	–	3		–	–	–	–	2	–	–	–	–	–		EPEC (3)
O?	1	–		–	–	–	–	5	–	–	–	–	–		NA (8)
Total	28	43		18	0	40	2	134	28	4	24	18	42		

a number in bracket represents the total of isolates.

b NA represents the isolates not allocated into pathotypes of EPEC, ETEC and STEC.

The *astA* gene and typical pathogenic *E. coli* (EPEC, ETEC, and STEC) from the farm-raised sika deer occurred more frequently than that of wild animals (including wide deer), and even than that of some domestic livestock. The *astA* gene encodes the toxin EAST1, which is associated with diarrhoea of postweaning pigs [5], [17]. Among the *E. coli* isolates from farm-raised sika deer, a high frequency of 60.9% was observed for the *astA* gene. The frequency of the gene *eaeA* (expressing the virulence of EPEC) was 19.5% in the obtained *E. coli* isolates, in comparison with 1.4% and 0.9% for Danish and Slovakia postweaning diarrhea pigs, respectively [5], [12]. Fifty-one isolates (23.2% of total obtained) contained *stx1* and/or *stx2* genes, in contrast with lower frequencies (16.3% and 10.5% ) of *stx*-gene containing *E. coli* isolates obtained from wild deer [18], [19]. The prevalence rates of the EPEC (20.9%) and STEC (23.2%) strains in this study were much higher than those (1.5% for EPEC and 5.5% for STEC) of wildlife in the south Belgium [7]. ETEC causes travelers diarrhoea by producing different combinations of heat labile (LT) and heat stable (ST) enterotoxins. The prevalence rate of ETEC strains was 31.4% in this study, compared with that of 55.3% and 33.2% from diarrheal pigs and calves, respectively [20], [21].

### Antimicrobial resistance of the *E. coli* isolates from the farm-raised sika deer

All the obtained *E. coli* isolates showed resistance to at least one of the tested antimicrobials (Table 2). Two hundred and eight (94.5%) *E. coli* isolates were resistant to 4 or more antimicrobials and 170 (77.3%) *E. coli* isolates were resistant to 7 or more antimicrobials. Forty-eight (21.8%) *E. coli* isolates were capable of resisting 9 antimicrobials. The most frequently resisted antimicrobials were sulfadiazine, sulfanethazine, tetracycline, ampicillin, amoxicillin, chloramphenicol, gentamicin, and ceftriaxone, which were the main antimicrobials used for sika deer diarrhea treatment. These results suggested that the antimicrobials resistance of the *E. coli* might be derived from the antimicrobials overuse of their hosts.

**Table 2:** Antimicrobial resistant phenotypes of *E. coli* strains isolated from farm-raised sika deer (n = 220).

Antimicrobials Group	Specific list	MIC(µg/ml)				Number of resistant strains (%)
		Resistance breakpoint	Range	MIC 50%	MIC 90%	
Amino-glycosides	Amikacin	64	1–256	16	128	98 (44.5%)
	Gentamicin	16	0.125–512	8	256	124 (56.4%)
	kanamycin	64	0.5–128	4	64	44 (20.0%)
	Streptomycin	64	0.5–512	16	256	88 (40.0%)
	Spectinomycin	64	0.5–512	16	128	76 (34.5%)
Cephems (parental)	Ceftiofur	8	0.125–256	4	32	58 (26.4%)
	Ceftriaxone	64	1–512	16	128	106 (48.2%)
Fluoroquinolones	Ciprofloxacin	4	0.0625–512	1	32	75 (34.1%)
	Enrofloxacin	2	0.0625–512	1	32	81 (36.8%)
	Norfloxacin	16	0.25–512	1	32	77 (35.0%)
Folate pathway inhibitors	Sulfadiazine	512	8–512	>512	>512	197 (89.5%)
	Sulfamethazine	512	8–512	>12	>512	182 (82.7%)
Penicillins	Amoxicillin	32	1–512	128	512	149 (67.7%)
	Ampicillin	32	1–512	128	512	157 (71.4%)
Penicol	Chloramphenicol	32	1–512	128	512	143 (65.0%)
Tetracycline	Tetracycline	16	0.125–512	64	256	176 (80.0%)

Similar to the livestock in China, farm-raised sika deers are usually supplied with heavy antimicrobials (e.g., cephalosporin, fluoroquinolone, aminoglycoside and sulfonamides) for disease treatment. Nearly all of the *E. coli* isolates in this study resisted four or more antimicrobials, which was similar to that of food-producing animals reported in China and other countries [1], [22], [23], [24]. Due to less contacted with antimicrobials, much lower abundances (7.3% and 8.8%, respectively) of the *E. coli* isolates from deer in Norway and USA were resistant to one or more of antimicrobials [8], [25]. A few previous studies showed that more than 60% of the *E. coli* isolates from humans and food-producing animals in China were resistant to fluoroquinolone drugs [23], [26]. In this study, 34.1%, 36.8%, and 35.0% of the *E. coli* isolates resisted ciprofloxacin, enrofloxacin, and norfloxacin, respectively. However, fluoroquinolone resistance was relatively low in other countries. For example, only 2.9% of the *E. coli* isolates from Danish dogs were resistant to ciprofloxacin and 8.0% of the *E. coli* isolates from Korean pigs were resistant to enrofloxacin [27], [28]. The *E. coli* isolates from wild deer were very sensitive to cephems [7]. Whereas, the cephems resistance of *E. coli* isolates this study was even much higher (26.4–48.2%) than that of food–producing animals (8.4% and 6.3%) [29], [30]. These significant differences may be ascribed to the fact that the antimicrobials are frequently overused on the deer farms in China.

The pathotypes and antimicrobials resistant genotypes of the obtained *E. coli* isolates resisting tetracycline, sulfonamides, and streptomycin-spectinomycin are shown in Table 3. The genes of *aadA*, *tetA*, *strA*, *strB* and *sul2* were the dominant antimicrobial-resistant genotypes in this study. For the analysis of tetracycline-resistant genes, *tetA*, *tetB* and *tetC* were found in 72, 29, and 10 strains, respectively. In contrast, 86 tetracycline-resistant isolates did not carry any of the tested tetracycline-resistant genes, indicating that other tetracycline-resistant genes (e.g., *tetD*, *tetE* or *tetM*) might be present, or other novel genetic resistant determinants exist [31]. Interestingly, at least one of the tested tetracycline-resistant genes (e.g., *tetA*, *teB* or *teC*) was detected in 6 tetracycline susceptible isolates. For the sulfonamides resistance analysis, genes of *sul1*, *sul2*, and *sul3* were found in 12, 98, and 5 isolates, respectively. Similar to the results of the tetracycline-resistant genes, at least one of the sulfonamides resistant genes (e.g., *sul1*, *sul2* or *sul3*) was detected in 20 sulfonamides susceptible isolates. For the aminoglycoside resistance, genes of *aadA*, *strA*, and *strB* were found in 55, 114, and 123 isolates, respectively. Twenty-five aminoglycoside resistant isolates did not carry any of the tested aminoglycoside resistant genes. However, at least one of the aminoglycoside resistant genes (e.g., *strA*, *strB* or *aadA*) was detected in 73 streptomycin and spectinomycin susceptible isolates.

**Table 3:** Antimicrobial resistant genotypes and pathotypes of *E. coli* strains from farm-raised sika deer (n = 220).

antimicrobials Group	Resistant gene	EPEC	ETEC	STEC	Other	Number of resistant strains
tetracycline	*tetA*	16	22	21	13	72
	*tetB*	9	6	7	7	29
	*tetC*	1	4	4	3	10
sulfonamides	*sul1*	1	5	2	4	12
	*sul2*	23	30	20	25	98
	*sul3*	1	2	1	1	5
Streptonmycin/spectinomycin	*strA*	18	37	30	29	114
	*strB*	22	41	28	32	123
	*aadA*	17	2	17	19	55

It is notable that majority (187 of 220) of the obtained *E. coli* isolates carried at least one of antimicrobial resistant genes that encodes resistant phenotypes to tetracycline, sulfonamides and streptomycin/spectionomycin, corresponding to high abundances of the genes of *tetA*, *sul2* and *strA*/*strB*. The tetracycline resistance in *E. coli* isolates from farm-raised sika deer was mostly due to *tetA* and *teB,* and the frequency of *tetA* (32.7%) was obviously higher than *tetB* (13.2%). However, previous studies showed that the frequency of *tetB* (49.8%) was higher than *tetA* (24.0%) for the *E. coli* isolates from pigs raised under overuse of antimicrobials in China [32]. The sulfonamides resistant *E. coli* is generally attributed to the presence of *sul1*, *sul2* and/or *sul3* genes [33]. The *sul2* gene displayed much higher frequency (44.5%) than that of the *sul1* and *sul3* (5.5% and 2.3%, respectively) in the *E. coli* isolates from this study. Other studies showed that the genes of *sul1*, *sul2* and *sul3* showed equal importance for sulfonamides-resistance in *E. coli* strains from food-producing animals in China [34], [35]. Among streptomycin/spectionomycin resistant genes, the *strA* and *strB* genes were detected at the highest frequency (51.8% and 55.9%, respectively). One previous study indicated that the *strA* and *strB* genes might be present together to make *E. coli* strains streptomycin resistance [36]. In addition, twelve streptomycin/spectionomycin susceptible *E. coli* isolates carried *aadA*, and the findings are consistent with former studies in which a large reservoir of nonintegrated gene cassettes could exist, but might not be expressed in some streptomy/spectionomycin sensitive *E. coli* strains [20], [36].

### Correlations between resistant and virulence genes in the farm-raised sika deer

Significant correlations (P<0.05) were found between a few virulence and resistant genes (Table 4). For example, the correlation coefficients between the resistant gene *sul2* and virulence genes of *aidA*, *elt* and *stx1* were 0.350, 0.318, and −0.400, respectively. The correlation coefficient was 0.333 between the resistant gene *strA* and the virulence gene *aidA*, and 0.316 between the resistant gene *aadA* and the virulence gene *fedA*. Besides of the above genes, no significant correlation was observed between the remaining resistant genes *(tetA*, *tetB*, *tetC*, *sul1*, *sul3* and *strB*) and the virulence genes (*eaeA*, *faeG*, *fasA*, *astA*, *estA*, *estB* and *stx2*). Such weak correlations between the selected resistant and virulence genes suggested that the presence of some virulence genes does not necessarily possess resistant characteristics for the *E. coli* of farm-raised sika deer. The results here was inconsistent with traditional view that frequent exposure to heavy antimicrobials might drive the distribution, reassortment and co-location of both resistant and virulence genes onto conjugative plasmids or pathogenicity islands in the pathogens, and the antimicrobial-resisting bacteria are more frequent as the pathogens than that of commensal bacteria [20]. Therefore, other factors may responsible for the observed prevalence and the associations of antimicrobial resistant and virulence genes.

**Table 4:** Pairwise statistical associations between antimicrobial resistant genes and virulence genes.

		*aidA*	*eaeA*	*faeG*	*fasA*	*fedA*	*astA*	*elt*	*estA*	*estB*	*stx1*	*stx2*
*tetA*	r	–0.070	0.107	(a)	–0.070	–0.171	(a)	–0.061	–0.015	0.072	–0.015	0.105
	p-value	0.668	0.512		0.668	0.291	0.000	0.711	0.928	0.658	0.928	0.520
*tetB*	r	–0.126	0.158	(a)	0.126	–0.154	(a)	0.044	–0.159	–0.087	–0.159	–0.126
	p-value	0.439	0.329		0.439	0.342	0.000	0.789	0.328	0.595	0.328	0.439
*tetC*	r	(a)	(a)	(a)	(a)	(a)	(a)	(a)	(a)	(a)	(a)	(a)
	p-value											
*sul1*	r	–0.095	–0.153	(a)	–0.095	–0.039	(a)	–0.274	0.146	–0.065	0.146	0.221
	p-value	0.560	0.345		0.560	0.812	0.000	0.087	0.368	0.689	0.368	0.170
*sul2*	r	0.350*	–0.033	(a)	0.017	0.143	(a)	0.318*	–0.259	0.011	–0.400*	0.184
	p–value	0.027	0.840		0.919	0.378	0.000	0.046	0.106	0.944	0.011	0.257
*sul3*	r	(a)	(a)	(a)	(a)	(a)	(a)	(a)	(a)	(a)	(a)	(a)
	p–value											
*strA*	r	0.333*	–0.060	(a)	0.167	–0.204	(a)	0.000	0.140	0.000	0.000	0.000
	p-value	0.036	0.714		0.304	0.206	0.000	1.000	0.389	1.000	1.000	1.000
*strB*	r	0.313	0.263	(a)	–0.312	0.040	(a)	0.225	–0.107	–0.018	–0.260	0.143
	p-value	0.052	0.106		0.053	0.808	0.000	0.168	0.519	0.914	0.109	0.384
*aadA*	r	0.086	0.077	(a)	–0.086	–0.316*	(a)	–0.030	0.253	0.059	0.253	0.086
	p-value	0.597	0.635		0.597	0.047	0.000	0.855	0.115	0.717	0.115	0.597

* Correlation is significant at the 0.05 level (p-value).

(a) The value cannot be calculated, because at least one of the variables is constant.

## Conclusions

Antimicrobials are used heavily for the farm-raised sika deer when disease outbreaks. The results of this study indicated that the pathogenic groups (EPEC, ETEC and STEC) of *E. coli* strains occurred at much higher frequency than that of wild life (including wild deer), and even higher than that of livestock in some developed countries. Furthermore, the antimicrobial resistance of the *E. coli* strains from farm-raised sika deer was also significantly higher than those reported in wild animals and certain livestock in some countries. Other than domestic livestock, antimicrobial-resisting *E. coli* strains from domesticated wildlife have become a new heavy contamination source, and already posed high potential risks to public health in Northeastern China. Feasible measurements should be taken for prudently antimicrobials use in domesticated wildlife and livestock to prevent the increasingly antimicrobial resistance problem from worsening. In addition, comprehensive surveys on domesticated wildlife for the antimicrobial-resisting bacteria are also strongly recommended to ensure the safety of food products and environments.

## Materials and Methods

### Ethics Statement

The fecal samples of the present study were collected from sika deer farms, no specific permissions were required for these locations/activities, the sika deer farms are the public open place in China, and the sample activities did not involve any endangered or protected species. This study focuses on the microbial antimicrobial-resistant characteristics of fecal samples from farm-raised sika deer, and the vertebrate materials are not included.

### Sample collection and *E. coli* strain isolation

**Figure 2:**
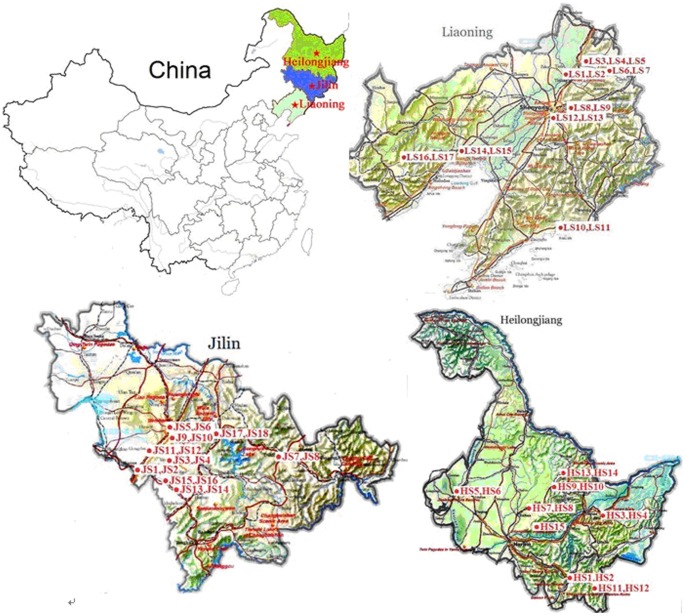
A map that shows 50 sika deer farms located in three Northeastern provinces of China. Note: symbols of LS, JS and HS represent the sample sites located in Liaoning, Jilin and Heilongjiang Provinces in China, respectively. Sites LS1, LS2 are located in Tieling; LS3, LS4, LS5 are located in Xifeng; LS6, LS7 are located in Qingyuan; LS8, LS9 are located in Fushun; LS10, LS11 are located in Dandong; LS12, LS13 are located in Shenyang; LS14, LS15 are located in Linghai; LS16, LS17 are located in Chaoyang; JS1, JS2 are located in Siping; JS3, JS4 are located in Yitong; JS5, JS6 are located in Changchun; JS7, JS8 are located in Dunhua; JS9, JS10 are located in Shuangyang; JS11, JS12 are located in Gongzhuling; JS13, JS14 are located in Dongfeng; JS15, JS16 are located in Liaoyuan; JS17, JS18 are located in Jilin, HS1, HS2 are located in Mudanjiang; HS3, HS4 are located in Shuangheshan; HS5, HS6 are located in Qiqihaer; HS7, HS8 are located in Shuiling; HS9, HS10 are located in Yichun; S11, HS12 are located in Dongning; HS13, HS14 are located in Wumahe; HS15 is located in Mulan.

*E. coli* isolates of this study were obtained from farm-raised sika deer with clinic signs of yellow/white diarrhea. All fecal samples were collected from 50 sika deer farms located in three Northeastern provinces of China, including 18 farms from Jilin province, 15 farms from Heilongjiang province and 17 farms from Liaoning province during the period of March to October in 2009 (Figure 2). In China, the deer farms are relatively small and usually raise less than 20 sika deers in each farm. One diarrheic fecal sample was collected from one location, and the antimicrobials usage information was obtained from the owners and from the medical records in each farm. The information of the antimicrobials usage in the studied farms within the last 12 months before sampling is shown in Table S1 and Fig. S1. The antimicrobials of sulfonamides, tetracycline and amino-glycosides were the most frequently used drugs for sika deer diarrhea treatment, followed by chloramphenicol, penicillins, cephems and fluoroquinolones (Fig. S1). Five types of antimicrobials (sulfonamides, tetracycline, amino-glycosides, chloramphenicol and penicillins) javascript:;were used in more than half of the investigated farms (Fig. S1).

All fecal samples were kept in dark, transported to the laboratory on ice, and analyzed for *E. coli* strains within 24 hrs. The fecal material was suspended in 0.9% (w/v) sterile NaCl (physiological salinity), spread on Petri dishes containing MacConkey agar (Oxoid, UK), followed by incubation at 37°C for 18–24 hrs [37]. Bacterial colonies with morphology of *E. coli* were randomly picked and identified by biochemical methods (Gram reaction; activities of catalase, oxidase and urease; indole production, methyl red reaction, Voges-Proskauer test and citrate utilization), and by the API 20E strips (BioMérieux, France) according to the manufacture instructions. The isolates identified as *E. coli* were maintained on Luria-Bertani (LB, Oxoid) slants at 4°C, and as glycerol suspension (20%, v/v) at –80°C for long-term preservation.

### Determination of O serogroups of *E. coli* isolates

Sika deer *E. coli* isolates proliferated on a nutrient agar (NA, BD) were suspended in 0.9% (w/v) NaCl, and then autoclaved at 1.05 kg f/cm^2^ for 1 h to extract somatic antigens. The serogroups of the *E. coli* isolates were examined by slide agglutination using O antisera commercially available from China Institute of Veterinary Drugs Control (IVDC, Beijing), and a NaCl control was performed to eliminate false positive results. Positive reactors were confirmed by tube agglutination test [38].

### Antimicrobial susceptibility test

Minimal inhibitory concentration (MIC) determination was performed by using the broth micro-dilution method according to the Clinical and Laboratory Standards Institute (CLSI, formerly NCCLS) Guideline (CLSI, 2009). The following 16 antimicrobials were selected for susceptibility test: amikacin, gentamicin, kanamycin, streptomycin, spectinomycin, ceftiofur, ceftriaxone, ciprofloxacin, enrofloxacin, norfloxacin, sulfadiazine, sulfamethazine, amoxicillin, ampicillin, chloramphenicol and tetracycline. The breakpoints for each antimicrobial resistance were outlined in table 2 according to the CLSI guidelines (CLSI, 2009). The isolates showing resistance to one or more antimicrobials were characterized for antibiotic resistant genes. Strain *E. coli* ATCC 25922 was applied as quality control for the susceptibility testing procedure.

### Polymerase chain reaction (PCR) detection of virulence genes in *E. coli* isolates

The PCR was applied to detect whether the obtained *E. coli* isolates harboring toxins (LT, STa, STb, Stx1, Stx2, and EAST1) and adhesions (F4, F5, F6, F18, AIDA, and EaeA), known as virulence genes for *E. coli* pathotypes causing intestinal diseases in humans and animals. The information of specific oligonucleotide primers, amplicons predicted sizes and pathotypes definition (EPEC, STEC, ETEC and EHEC) for the tested virulence genes are shown in Table 5. The PCR conditions for each virulence gene were performed as described previously [39], [40], [41], [42]. Three strains from Zhejiang Province Key Laboratory for Food Safety were selected as positive controls for determining pathotypes of EPEC, ETEC and STEC.

**Table 5:** Primers and the predicted size of the virulence genes associated with different *E. coli* pathotypes.

Virulence factors	*E. coli* pathotypes	Description/function	Primers	Primer sequences	predicted sizes	Reference
LT (*elt*)	ETEC	Heat-labile toxin	elt-F	GGC GTT ACT ATC CTC TCT AT	272	[39]
			elt-R	TGG TCT CGG TCA GAT ATG T		
STa (*estA*)	ETEC	Heat-stable enterotoxin a	estA-F	CAA CTG AAA TCA CTT GAC TCT T	158	[39]
			estA-R	TTA ATA ACA TCC AGC ACA GG		
STb (*estB*)	ETEC	Heat-stable enterotoxin b	estB-F	TGC CTA TGC ATC TAC ACA AT	113	[39]
			estB-R	CTC CAG CAG TAC CAT CAC CTA		
Stx1 (*stx1*)	STEC (EHEC)	Shiga toxin I	stx1-F	CGC TGA ATG TCA TTC GCT CTG C	302	[40]
			stx1-R	CGT GGT ATA GCT ACT GTC ACC		
Stx2 (*stx2*)	STEC (EHEC)	Shiga toxin II	stx2-F	CTT CGG TAT CCT ATT CCC GG	516	[40]
			stx2-R	CTG CTG TGA CAG TGA CAA AAC GC'		
EAST1 (*astA*)	EaggEC	EaggEC (heat-stable enterotoxin)	astA-F	TCG GAT GCC ATC AAC ACA GT	125	[41]
			astA-R	GTC GCG AGT GAC GGC TTT GTA AG		
F4 (*faeG*)	ETEC	Fimbrial adhesin	faeG-F	GAA TCT GTC CGA GAA TATCA	499	[39]
			faeG -R	GTT GGT ACA GGT CTT AAT GG		
F5 (*fanC*)	ETEC	Fimbrial adhesin	fanC -F	TGC GAC TAC CAA TGC TTC TG	450	[42]
			fanC -R	TAT CCA CCA TTA GAC GGA GC		
F6 (*fasA*)	ETEC	Fimbrial adhesin	fasA-F	TCT GCT CTT AAA GCT ACT GG	333	[39]
			fasA-R	AAC TCC ACC GTT TGT ATC AG		
F18 (*fedA*)	ETEC	Fimbrial adhesin	fedA-F	TGG TAA CGT ATC AGC AAC TA	313	[39]
			fedA-R	ACT TAC AGT GCT ATT CGA CG		
AIDA (*aidA-1*)	EPEC/DAEC	Adhesin involved in diffuse adherence	aidA- F	ACA GTA TCA TAT GGA GCC A	585	[41]
			aidA-R	TGT GCG CCA GAA CTA TTA		
EaeA (*eaeA*)	EPEC/EHEC	Intimin	eae-F	GGA ACG GCA GAG GTT AAT CTG CAG	775	[40]
			eae-R	GGC GCT CAT CAT AGT CTT TC		

Note: DAEC refers to diffusely adherent *E. coli*; EaggEC refers to Enteroaggregative *E. coli*; EHEC refers to Enterohemorrhage *E. coli*.

### Antimicrobial-resisting gene detection

Antimicrobial-resisting genes were detected and identified following the protocols as described previously (Table 6). Briefly, the *E. coli* strains were grown in 500 µl LB broth overnight, and 20 µl of the culture was transferred to 200 µl lysis buffer [0.1 M Tris-HCl (pH 8.5), 0.05% Tween 20, and 0.24 mg/ml proteinase K]. The sample was incubated at 60°C for 1 hour and subsequently heated at 97°C for 15 min. The PCR primers and annealing temperatures for major resisting genes of tetracycline (*tetA*, *tetB* and *tetC*), sulfonamides (*sul1*, *sul2* and *sul3*), and streptomycin-spectinomycin (*strA*/*strB* and *aadA*) were detailed in Table 6, and the major resisting genes were amplified by a set of multiplex PCR protocols [36], [43], [44]. The multiplex PCRs were all performed with a total 25-µl reaction mixture and a Qiagen multiplex PCR kit (Qiagen, Shanghai) with 1 µl Qiagen multiplex PCR master mixture, 1× Q-solution, and 1× primer mixture according to the manufacturer's instructions. The PCRs were performed as follows: 1 cycle of 4 min at 95°C; 35 cycles, each consisting of 1 min at 95°C, 1 min at annealing temperature, and 1 min at 72°C; and 1 cycle of 7 min at 72°C.

**Table 6:** Primers and single PCR conditions of the 9 resistant genes.

Gene	Primer name	Oligonucleotide sequences of primers	Annealing (°C)	Amplified Products (bp)	Reference
*tetA*	tetA-F	GGC GGT CTT CTT CAT CAT GC	64	502	[36]
	tetA-R	CGG CAG GCA GAG CAA GTA GA			
*tetB*	tetB-F	CAT TAA TAG GCG CAT CGC TG	64	930	[36]
	tetB-R	TGA AGG TCA TCG ATA GCA GG			
*tetC*	tetC-F	GCT GTA GGC ATA GGC TTG GT	64	888	[36]
	tetC-R	GCC GGA AGC GAG AAG AAT CA			
*sul1*	sul1-F	GTG ACG GTG TTC GGC ATT CT	68	779	[36]
	sul1-R	TCC GAG AAG GTG ATT GCG CT			
*sul2*	sul2-F	CGG CAT CGT CAA CAT AAC CT	66	721	[36]
	sul2-R	TGT GCG GAT GAA GTC AGC TC			
*sul3*	sul3-F	GAG CAA GAT TTT TGG AAT CG	51	880	[43]
	sul3-R	CAT CTG CAG CTA ACC TAG GGC TTT GGA			
*strA*	strA-F	CCT GGT GAT AAC GGC AAT TC	55	546	[44]
	strA-R	CCA ATC GCA GAT AGA AGG C			
*strB*	strB-F	ATC GTC AAG GGA TTG AAA CC	55	509	[44]
	strB-R	GGA TCG TAG AAC ATA TTG GC			
*aadA*	aadA-F	GTG GAT GGC GGC CTG AAG CC	68	525	[44]
	aadA-R	AAT GCC CAG TCG GCA GCG			

### Statistical analysis

For the purpose of statistical analysis, isolates with reduced susceptibility were classified into resistant groups. The pairwise statistical associations between major antimicrobial-resisting and virulence genes were determined by using the Statistical Package for the Social Sciences (SPSS version 13.0; SPSS, Chicago, IL, USA).

## Supporting Information

Figure S1 **Frequency of antimicrobials usage in 50 sika deer farms.** **Note**: Antimicrobials abbreviations are the same as Table S1.
(TIF)

Table S1 **Antimicrobials usage information in 50 sample locations. Antimicrobials abbreviations:** AMI, Amikacin; EN, Gentamicin; KAN, Kanamycin; STR, Streptomycin; SPE, Spectinomycin; CER, Ceftiofur; CEE, Ceftriaxone; CIP, Ciprofloxacin; ENR, Enrofloxacin; NOR, Norfloxacin; SDM, Sulfadiazine; SMZ, Sulfamethazine; AMO, Amoxicillin; AMP, Ampicillin; CHL, Chloramphenicol; TET, Tetracycline; **Note:** •, the antimicrobial was used; ○, the antimicrobial was not used. **Note:** Sample locations of LS, JS and HS are the same as Figure 2.
(DOC)
