# Supplementary figures and images for: Genotypic and Phenotypic Characterization of Antimicrobial-Resistant Escherichia coli from Farm-Raised Diarrheic Sika Deer in Northeastern China
Source: PLoS One. 2013 Sep 9;8(9):e73342. doi: 10.1371/journal.pone.0073342 (PMC3767801; doi:10.1371/journal.pone.0073342)

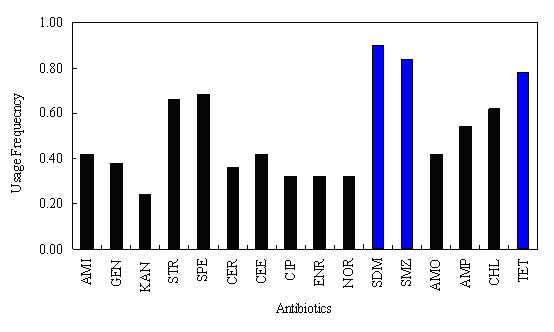

Supplement: Figure S1 — Frequency of antimicrobials usage in 50 sika deer farms. Note: Antimicrobials abbreviations are the same as Table S1. (TIF) [file pone.0073342.s001.tif]
