# Supplementary material for: Genotypic and Phenotypic Characterization of Antimicrobial-Resistant Escherichia coli from Farm-Raised Diarrheic Sika Deer in Northeastern China
Source: PLoS One. 2013 Sep 9;8(9):e73342. doi: 10.1371/journal.pone.0073342 (PMC3767801; doi:10.1371/journal.pone.0073342)
Supplement: Table S1 — Antimicrobials usage information in 50 sample locations. Antimicrobials abbreviations: AMI, Amikacin; EN, Gentamicin; KAN, Kanamycin; STR, Streptomycin; SPE, Spectinomycin; CER, Ceftiofur; CEE, Ceftriaxone; CIP, Ciprofloxacin; ENR, Enrofloxacin; NOR, Norfloxacin; SDM, Sulfadiazine; SMZ, Sulfamethazine; AMO, Amoxicillin; AMP, Ampicillin; CHL, Chloramphenicol; TET, Tetracycline; Note: •, the antimicrobial was used; ○, the antimicrobial was not used. Note: Sample locations of LS, JS and HS are the same as Figure 2. (DOC) [file pone.0073342.s002.doc]

| Sample location | AMI | GEN | KAN | STR | SPE | CER | CEE | CIP | ENR | NOR | SDM | SMZ | AMO | AMP | CHL | TET |
| --- | --- | --- | --- | --- | --- | --- | --- | --- | --- | --- | --- | --- | --- | --- | --- | --- |
| LS1 | ● | ● | ● | ● | ● | ● | ● | ● | ○ | ● | ● | ● | ● | ○ | ● | ● |
| LS2 | ● | ○ | ○ | ● | ○ | ● | ○ | ○ | ● | ○ | ● | ○ | ○ | ● | ● | ● |
| LS3 | ○ | ● | ● | ● | ● | ● | ● | ● | ● | ● | ● | ● | ● | ○ | ● | ● |
| LS4 | ● | ○ | ○ | ○ | ● | ○ | ● | ○ | ○ | ● | ● | ● | ● | ● | ● | ● |
| LS5 | ○ | ○ | ● | ● | ● | ○ | ○ | ● | ● | ○ | ● | ● | ○ | ● | ● | ● |
| LS6 | ● | ○ | ○ | ● | ● | ○ | ○ | ● | ○ | ● | ● | ● | ● | ● | ● | ○ |
| LS7 | ○ | ● | ○ | ● | ● | ● | ● | ○ | ● | ○ | ○ | ● | ● | ● | ● | ● |
| LS8 | ○ | ● | ○ | ● | ○ | ○ | ○ | ○ | ● | ○ | ● | ● | ○ | ● | ● | ● |
| LS9 | ○ | ○ | ○ | ○ | ● | ● | ● | ● | ○ | ● | ● | ● | ○ | ○ | ● | ● |
| LS1 | ○ | ○ | ● | ● | ● | ○ | ○ | ○ | ○ | ○ | ● | ● | ○ | ○ | ● | ● |
| LS11 | ● | ○ | ○ | ○ | ● | ○ | ● | ● | ○ | ○ | ● | ● | ● | ○ | ○ | ● |
| LS12 | ● | ○ | ○ | ● | ○ | ○ | ○ | ○ | ○ | ○ | ● | ● | ● | ○ | ● | ● |
| LS12 | ○ | ● | ○ | ○ | ○ | ○ | ● | ● | ○ | ○ | ● | ● | ○ | ● | ● | ● |
| LS14 | ● | ○ | ○ | ● | ● | ○ | ○ | ○ | ○ | ● | ● | ○ | ● | ● | ● | ○ |
| LS15 | ○ | ○ | ● | ● | ● | ● | ○ | ○ | ● | ○ | ● | ● | ● | ○ | ○ | ● |
| LS16 | ● | ○ | ○ | ● | ● | ○ | ● | ○ | ○ | ● | ● | ● | ○ | ● | ● | ● |
| LS17 | ○ | ● | ○ | ○ | ○ | ● | ○ | ○ | ○ | ○ | ○ | ● | ○ | ● | ● | ○ |
| JS1 | ○ | ○ | ○ | ● | ● | ○ | ○ | ● | ○ | ○ | ● | ○ | ○ | ○ | ● | ● |
| JS2 | ● | ● | ○ | ● | ○ | ● | ● | ○ | ● | ● | ● | ● | ● | ● | ● | ● |
| JS3 | ○ | ○ | ● | ○ | ● | ○ | ○ | ○ | ○ | ○ | ○ | ● | ○ | ● | ● | ● |
| JS4 | ● | ● | ○ | ○ | ● | ● | ○ | ● | ● | ○ | ● | ○ | ● | ○ | ● | ● |
| JS5 | ○ | ● | ○ | ● | ○ | ● | ● | ○ | ● | ○ | ● | ● | ● | ● | ○ | ● |
| JS6 | ○ | ○ | ● | ● | ● | ○ | ○ | ○ | ○ | ○ | ● | ● | ○ | ● | ○ | ● |
| JS7 | ● | ○ | ○ | ○ | ○ | ● | ○ | ● | ● | ○ | ● | ○ | ● | ● | ○ | ● |
| JS8 | ○ | ● | ○ | ○ | ● | ○ | ○ | ○ | ○ | ● | ● | ● | ● | ○ | ○ | ● |
| JS9 | ● | ○ | ○ | ● | ○ | ○ | ● | ○ | ● | ○ | ● | ● | ○ | ○ | ○ | ● |
| JS10 | ○ | ● | ○ | ● | ● | ● | ○ | ○ | ○ | ● | ○ | ● | ● | ● | ● | ○ |
| JS11 | ● | ○ | ○ | ● | ● | ○ | ○ | ● | ○ | ○ | ● | ● | ○ | ● | ● | ● |
| JS12 | ○ | ● | ○ | ○ | ● | ○ | ● | ○ | ○ | ○ | ● | ● | ○ | ○ | ○ | ● |
| JS13 | ○ | ○ | ○ | ● | ○ | ○ | ○ | ○ | ● | ○ | ● | ● | ○ | ● | ● | ○ |
| JS14 | ● | ● | ○ | ● | ● | ○ | ○ | ○ | ○ | ○ | ● | ● | ● | ● | ○ | ● |
| JS15 | ○ | ○ | ○ | ○ | ○ | ○ | ● | ○ | ○ | ● | ○ | ● | ● | ○ | ● | ● |
| JS16 | ○ | ○ | ○ | ● | ● | ● | ○ | ○ | ○ | ○ | ● | ● | ○ | ● | ○ | ○ |
| JS17 | ● | ● | ○ | ● | ● | ○ | ○ | ● | ○ | ○ | ● | ● | ● | ● | ● | ● |
| JS18 | ○ | ○ | ○ | ○ | ○ | ● | ● | ○ | ○ | ● | ● | ● | ○ | ○ | ● | ○ |
| HS1 | ● | ● | ● | ● | ● | ○ | ○ | ○ | ○ | ○ | ● | ○ | ● | ○ | ○ | ● |
| HS2 | ○ | ○ | ○ | ● | ● | ● | ● | ○ | ● | ○ | ● | ● | ● | ○ | ● | ● |
| HS3 | ○ | ○ | ○ | ○ | ○ | ○ | ● | ○ | ○ | ○ | ● | ● | ○ | ○ | ● | ● |
| HS4 | ● | ● | ○ | ● | ● | ○ | ○ | ● | ● | ○ | ● | ● | ● | ○ | ○ | ● |
| HS5 | ● | ○ | ○ | ○ | ● | ○ | ● | ○ | ○ | ○ | ● | ● | ○ | ○ | ○ | ○ |
| HS6 | ○ | ● | ○ | ● | ● | ○ | ○ | ○ | ○ | ● | ● | ● | ○ | ● | ● | ○ |
| HS7 | ○ | ○ | ○ | ● | ● | ● | ● | ● | ● | ○ | ● | ○ | ○ | ● | ○ | ○ |
| HS8 | ○ | ○ | ● | ○ | ● | ○ | ○ | ○ | ○ | ○ | ● | ● | ○ | ○ | ○ | ● |
| HS9 | ● | ○ | ○ | ● | ○ | ○ | ○ | ○ | ○ | ● | ● | ● | ○ | ● | ○ | ● |
| HS10 | ○ | ○ | ● | ● | ● | ● | ○ | ○ | ○ | ○ | ● | ○ | ○ | ○ | ● | ● |
| HS11 | ○ | ○ | ○ | ● | ○ | ○ | ● | ● | ○ | ○ | ● | ● | ○ | ● | ○ | ○ |
| HS12 | ● | ● | ● | ○ | ● | ○ | ○ | ○ | ○ | ● | ● | ● | ○ | ● | ● | ● |
| HS13 | ○ | ○ | ○ | ● | ● | ● | ● | ○ | ○ | ○ | ● | ● | ○ | ● | ● | ● |
| HS14 | ● | ● | ● | ● | ● | ○ | ○ | ○ | ○ | ● | ● | ● | ○ | ○ | ○ | ● |
| HS15 | ○ | ○ | ○ | ○ | ○ | ○ | ● | ● | ● | ○ | ● | ● | ○ | ○ | ○ | ● |
